# Pheromone Binding Protein EhipPBP1 Is Highly Enriched in the Male Antennae of the Seabuckthorn Carpenterworm and Is Binding to Sex Pheromone Components

**DOI:** 10.3389/fphys.2018.00447

**Published:** 2018-04-27

**Authors:** Ping Hu, Chenglong Gao, Shixiang Zong, Youqing Luo, Jing Tao

**Affiliations:** ^1^Beijing Key Laboratory for Forest Pest Control, Beijing Forestry University, Beijing, China; ^2^Xing An Vocational and Technical College, Xinganmeng, China

**Keywords:** *Eogystia hippophaecolus*, pheromone-binding protein, real-time PCR, western blot, fluorescence competitive binding assay

## Abstract

The seabuckthorn carpenterworm moth *Eogystia hippophaecolus* is a major threat to seabuckthorn plantations, causing considerable ecological and economic losses in China. Transcriptomic analysis of *E. hippophaecolus* previously identified 137 olfactory proteins, including three pheromone-binding proteins (PBPs). We investigated the function of *E. hippophaecolus* PBP1 by studying its mRNA and protein expression profiles and its binding ability with different compounds. The highest levels of expression were in the antennae, particularly in males, with much lower levels of expression in the legs and external genitals. Recombinant PBP1 showed strong binding to sex-pheromone components, suggesting that antennal EhipPBP1 is involved in binding sex-pheromone components during pheromone communication.

## Introduction

Recognition of sex pheromones facilitates sexual reproduction and species-specific reproductive isolation. By now identified pheromone-binding proteins (PBPs), chemosensory proteins (CSPs) and membrane-bound proteins such as odorant receptors (ORs), ionotropic receptors (IRs), Gustatory receptors (GRs) and sensory neuron membrane proteins (SNMPs) are involved in insect olfactory recognition (Leal, 2013). PBPs are a subtype of odorant-binding proteins (OBPs) with a major function of pheromone binding, and act to bind and deliver pheromones to their relevant ORs, or PBP. Pheromone complex activate ORs (Vogt and Riddiford, 1981; Krieger et al., 1996; Zhou, 2010; Leal, 2013).

Pheromone-binding proteins are small, water-soluble, extracellular proteins of around 130–150 amino acids, with molecular weights of 15–20 kDa, containing six or seven alpha helices that form a conical binding cavity, and six cysteine residues that form three disulfide bonds, maintaining the stability of the three-dimensional structure (Leal et al., 1999; Scaloni et al., 1999). PBP expression has mostly been identified in male antennae, but lower levels of expression have also been found in female antennae, for example, in *Manduca sexta* (Gyorgyi et al., 1988; Vogt et al., 2002), *Spodoptera exigua* (Xiu and Dong, 2007) and *Cydia pomonella* (Tian et al., 2016). As the research further develops, PBP were identified and located in the antennal long trichoid sensilla of *Sesamia nonagrioides* (De et al., 2006), *Bombyx mori* and *Antheraea polyphemus* (Steinbrecht et al., 1995). Apart from the antennae, PBPs have been identified in appendages such as the proboscis, labipalps, and legs (Zhang et al., 2013; De Biasio et al., 2014), and also in the sex pheromone gland of *Heliothis virescens* (Widmayer et al., 2009; Vogel et al., 2010) and *Agrotis ipsilon* (Gu et al., 2013).

Specific pheromone binding by PBPs has been demonstrated in PBP1 and PBP2 of *A. polyphemus* (Pophof, 2002, 2004), PBP2 of *H. virescens* (Grosse-Wilde et al., 2007), PBP1 and PBP2 of *A. pernyi* (Guérin-Méneville) (Du and Prestwich, 1995; Maida et al., 2003), and PBP1 and PBP2 of *Lymantria dispar* (Plettner et al., 2000), all of which can bind a pheromone component selectively. Other results have demonstrated PBPs binds pheromone components without specificity. For example, PBP1 of *Mamestra brassicae* bind all its pheromone components, (*Z*)-11-hexadecenal (Z11-16: Ald), (*Z*)-11-hexadecenol (Z11-16:OH), and (*Z*)-11-hexadecenyl acetate (Z11-16:OAc) (Campanacci et al., 2001). PBP1 of *Amyelois transitella* (Walker) can bind two pheromone compounds, (11Z,13Z)-hexadecadienal (Z11,Z13-16:Ald) and (11Z,13Z)-hexadecadien-1-ol (Z11,Z13-16:OH), and the behavioral antagonist (11Z,13Z)-hexadecadien-1-yl acetate (Z11,Z13-16:OAc) (Leal et al., 2009). Three PBPs of *Plutella xylostella* (L.) do not show preferential binding to any individual component of four pheromone components, and also bind pheromone-component analogs (Sun et al., 2013). Besides, PBPs also bind to plant volatiles. For instance, PBP3 of *P. xylostella* has weak affinities for all tested plant volatiles (Sun et al., 2013), and PBP1 of *M. sexta* binds fatty acids, especially palmitic acid (Campanacci et al., 2001). In the PBPs binding ability reports, about 60% of the PBPs display no specificity (Liu et al., 2014). With regard to the mechanism of PBP ligand binding and release, pH has been shown to change the conformation of PBPs. At low pH, the C terminus of the PBP forms an α-helix, accelerating release of ligand from the binding cavity. At physiological pH, the C terminus of the PBP releases the α-helix and the binding cavity opens, prompting PBP ligand binding. This mechanism has been verified in *B. mori* PBP1 (Wojtasek and Leal, 1999; Damberger et al., 2000; Sandler et al., 2000; Horst et al., 2001a,b; Lee et al., 2002; Leal et al., 2005). The proposed mechanism underlying the specificity of the insect sex pheromone-binding mechanism is as follow: PBPs can specifically combine with pheromone components, to function as an initial filter (Leal et al., 2005); the ORs may then only be activated by either a specific pheromone component or a pheromone component bound to a PBP. This mechanism combines the specificity of both PBPs and ORs, so that, even if the individual specificities are not strong, specific sex-pheromone binding can be maintained (Forstner et al., 2006, 2009; Grater et al., 2006).

The seabuckthorn carpenterworm *Eogystia hippophaecolus* (Lepidoptera: Cossidae) damages plantations of the seabuckthorn *Hippophae rhamnoides* L. (Rosales: *Elaeagnaceae*), which is widely distributed throughout “the Northwest-North-Northeast China Networks of Shelterbelts,” and functions as preventing soil erosion and desertification (Marchal et al., 2011). *E. hippophaecolus* also damages *Ulmus pumila* L. (Urticales: *Ulmaceae*), as well as several other species in the *Rosaceae* family (Zong et al., 2006). The pheromone compounds of the *E. hippophaecolus* female sex-pheromone gland have been identified as (*Z*)-7-tetradecenyl acetate (Z7-14:Ac), and (*E*)-3-tetradecenyl acetate (E3-14:Ac) (Fang et al., 2005; Zong et al., 2010), and have been used alone or with (E)-7- tetradecenyl acetate (E7-14:Ac) to develop specific and efficient artificial sex pheromone traps (Fang et al., 2005; Zong et al., 2010). Besides, 29 *E. hippophaecolus* OBP transcripts have been identified, including three PBPs, and phylogenetic analysis placed EhipPBP1 in the PBP-C sub-lineage, EhipPBP2 in the PBP-A sub-lineage, and EhipPBP3 in the PBP-D sub-lineage (Hu et al., 2016). However, it has not previously been determined whether *E. hippophaecolus* PBPs are expressed in the antennae at protein level, and could fulfill olfactory functions in *E. hippophaecolus*.

Considering that EhipPBP1 was highest expressed in the antennae (Hu et al., 2016), in the current study, we focused on the tissue distribution and ligand binding of EhipPBP1, to provide information on its function.

## Materials and Methods

### Ethics Statement

The seabuckthorn carpenterworm *E. hippophaecolus* is a common forestry pest in China, the collection of which was made with the direct permission of the Jianping forest bureau. This species is not included in the “List of Endangered and Protected Animals in China.” All operations were performed according to ethical guidelines in order to minimize pain and discomfort to the insects.

### Insect and Tissue Collection

*Eogystia hippophaecolus* were collected from a damaged seabuckthorn forest using light and sex pheromone traps from the middle of June to the end of July in 2014 and 2015 in Jianping, Liaoning, China. Antennae, legs (propodeums, mesopodiums, metapedes), external genitals, labipalps from adult males and females were excised and stored in RNAlater (Ambion, Austin, TX, United States). Then all samples were taken back indoor and stored at -80°C.

### RNA Extraction and cDNA Synthesis

Total RNA was extracted from the antennae of 10 males and 10 females using TRIzol reagent (Ambion) and the RNeasy Plus Mini Kit (No. 74134; Qiagen, Hilden, Germany) following the manufacturer’s instructions. RNA quantity was detected using the NanoDrop 2008 (Thermo, Waltham, MA, United States). cDNA was synthesized from total RNA using the PrimeScript RT Reagent Kit with gDNA Eraser to remove gDNA (No. RR047A; TaKaRa, Shiga, Japan), and immediately used for PCR amplification or stored at -20°C until further use.

### Expression Analysis by Fluorescence Quantitative Real-Time PCR

Fluorescence quantitative real-time PCR was performed to examine the expression of EhipPBP1 in six tissues with chemosensory functions of females and males. Antennae, legs (including the propodeum, mesopodium, and metapedes), external genitals, labial palps were collected from ten female and male *E. hippophaecolus*, respectively, and total RNA of six tissues with chemosensory functions were extracted following the methods described above. The propodeum, mesopodium, and metapedes RNA were accounted for one third of all leg RNA. NanoDrop 2008 and agarose gel electrophoresis examined density and quality of RNA. cDNA Synthesis was performed as previously indicated. Gene-specific primers were designed using Primer3^1^. The sequence of the gene-specific primers of EhipPBP1 were as follow: forward primer: 5′-CGAATGCAAACAAGAGCTGA-3′; reverse primer: 5′- TTTGC GTTTCCATGGTGTAA-3′. According to Minimum Information for Publication of Quantitative Real-Time PCR Experiments (MIQE) (Bustin et al., 2009), an appropriate reference gene is fundamental for optimum qPCR analysis. The sequences of the actin gene primers were based on those reported in previous publication and were as follow: forward primer 5′-CGACT TCGAACAGGAGATGG -3′; reverse primer 5′- TCGTCTCATGAATGCCACAG -3′ (Hu et al., 2016). A PCR analysis was conducted using the Bio-Rad CFX96 PCR System (Hercules, CA, United States). SYBR Premix Ex Taq II (No. RR820A; TaKaRa) was used for the PCR reaction under a two-steps amplification. Each PCR reaction was conducted in a 25 μl reaction mixture containing 12.5 μl of SYBR Premix Ex Taq^TM^ II, 1 μl of each primer (10 mM), 2 μl of sample cDNA (2.5 ng of RNA), and 8.5 μl of dH_2_O (sterile distilled water). The RT-qPCR cycling parameters were as follow: 95°C for 30 s, followed by 40 cycles of 95°C for 5 s, 60°C for 30 s, and 65°C to 95°C in increments of 0.5°C for 5 s to generate the melting curves. To examine reproducibility, each qPCR reaction for each tissue was performed in three biological replicates and three technical replicates, in which each biological replication was with 10 individuals, each biological replication with three technical replicates. Negative controls without either template were included in each experiment. Bio-Rad CFX Manager (version 3.1.1517.0823) was used to normalize expression based on ΔΔ*C*q values, with female labial palps in analyze mode as control samples, and the 2^-ΔΔ^*^C^*^T^ method was used (the amplification efficiency of EhipPBP1 was equal to 100%) (Livak and Schmittgen, 2001). Before comparative analyses, we examined the normal distribution and performed an equal variances test to make sure the data followed a normal distribution and presented an equal variances. The results of comparative analysis of EhipPBP1 in four tissue types were assessed by a one-way nested analysis of variance (ANOVA), followed by Tukey’s honestly significance difference (HSD) tests implemented in SPSS Statistics 18.0 (IBM Corporation, United States) Values are presented as means ± SE.

### Cloning and Sequencing

Analysis of the antennal transcriptome of *E. hippophaecolus* indicates that the EhipPBP1 gene has an open reading frame (ORF) >400 bp that includes a sequence encoding a signal peptide, and so it is a complete gene (Hu et al., 2016). Part of the coding region (ORF) of EhipPBP1 was amplified by polymerase chain reaction (PCR) with the following gene-specific primers: forward primer, 5′-GGACAACTGCAA CTCTTTGTCG-3′; reverse primer, 5′-GAGACCACAGATGGTGATGAGC-3′ and cDNA of male antenna as template. Primers were designed to contain the full ORF sequence, so target band sequence contain all ORF and longer than ORF. PCR was performed using Ex Taq DNA polymerase (Takara, Dalian, China) with 34 cycles of 98°C for 10 s, 55°C for 50 s, and 72°C for 5 s. The PCR products were digested and ligated into the pEASY-T Easy Vector (TransGen, Beijing, China). The recombinant plasmid was transformed into *Escherichia coli* DH5α competent cells and plated onto LB agar medium containing ampicillin (1 ml LB: 1 μl ampicillin). Colony PCR was used to select positive clones and the amplified DNA was then sequenced (Qingke, Beijing, China).

### Sequences and Structural Analysis

From the coding sequence of EhipPBP1, the ORFs were deduced using the Open Reading Frame Finder^2^. Putative signal peptides were predicted using the SignalP 4.1 Server^3^ (Petersen et al., 2011). The molecular weights of the proteins were predicted using SWISS-PROT^4^. Three-dimensional models of EhipPBP1 were predicted using the SWISS MODEL online tools^5^ (Biasini et al., 2014). Template search with Blast (Altschul et al., 1997) and HHBlits (Remmert et al., 2011) in default parameters has been performed against the SWISS-MODEL template library (SMTL, last update: 2017-12-06, last included PDB release: 2017-12-01). The templates with the highest quality have then been selected for model building. Models are built based on the target-template alignment using ProMod3 (Guex and Peitsch, 1997) in default parameters. The rationale underlying the model evaluation was based on a Ramachandran plot (Ramachandran et al., 1963).

### Recombinant Expression and Purification

The coding sequence of EhipPBP1 (441 bp), omitting the sequence encoding the signal peptide was amplified using cDNA from male antennae with Ex Taq DNA polymerase (TaKaRa) by PCR using gene-specific primers containing the restriction enzyme sites *NdeI* in the forward primer (5′-**CATATG**GAGATAGATAGTTCAGC AGAAACAA-3′) and *BglII* in the reverse primer (5′-**AGATCT**TTACATTTCAGT AAGTACTTCAGTAACG-3′). The amplification conditions were 34 cycles of 98°C for 10 s, 55°C for 50 s, and 72°C for 5 s. After analysis on a 1.5% agarose gel, PCR product was purified with the Axygen Gel Extraction Kit (Axygen, NY, United States) and cloned into pEASY-T Easy Vector (Transgen, Beijing China). Positive clones were selected by PCR and sequenced. Plasmids were extracted with the Axyprep Plasmid Miniprep Kit (Axygen, NY, United States), and digested with *NdeI* and *BglII*, and the fragment encoding the correct EhipPBP1 sequence was purified and sub-cloned into the bacterial expression vector pET30a (+) (Novagen, Madison, WI, United States), and then verified by sequencing. Plasmids containing the correct insert (pET30a-EhipPBP1) were then transformed into *E. coli* BL21 (DE3) pLysS cells. Expression of EhipPBP1 was induced with isopropyl-β-D-thiogalactopyranoside (IPTG) at a final concentration of 1 mM at 37°C for 6 h. Samples were then sonicated and centrifuged with 6000 *g* at 4°C for 15 min, and the supernatant and pellet were analyzed by sodium dodecyl sulfate polyacrylamide-gel electrophoresis (SDS-PAGE). EhipPBP1 occurred as inclusion bodies in pellet, which were purified by Ni-ion affinity chromatography (Qiagen, Hilden, Germany). Soluble protein was obtained by denaturation of the inclusion bodies, followed by renaturation with 8–0.5 M urea renaturation buffer. The protein was concentrated by the use of Amicon Ultra concentrators with a 10 kDa cutoff (Millipore, Billerica, MA, United States), and purity was confirmed by SDS-PAGE analysis. The concentration of EhipPBP1 protein was measured by the Bradford method with BSA as the standard protein.

### Preparation of the Polyclonal Antibody

Polyclonal antibody were obtained by subcutaneous injection of adult rabbits with 300 μg of recombinant EhipPBP1 protein, followed by three additional injections of 250 μg on the 21st, 35th, and 49th day. Two rabbits were used in a parallel study. The proteins were emulsified with an equal volume of Freund’s complete adjuvant on the first injection and Freund’s incomplete adjuvant on the second and subsequent injections. The polyclonal sera was tested by enzyme-linked immunosorbent assay (ELISA). Rabbits were exsanguinated 10 days after the last injection, and then used Protein G (GE Healthcare, United States) purification sera to obtain polyclonal antibody.

### Western Blotting Analyses

Protein extracts were separately prepared from female and male *E. hippophaecolus* antennae, legs (extract propodeum, mesopodium, and metapedes protein first, then mixing one-third of them), external genitals, and labial palps. Protein concentrations were measured by the Bradford method with BSA as the standard protein (Bradford, 1976). After protein electrophoresis under denaturing conditions (15% SDS-PAGE), duplicate gels were prepared for analysis. One gel was stained with 0.1% Coomassie brilliant blue R-250 (in 10% acetic acid and 45% methanol), and proteins were transferred from the other gel by transfer membrane electrophoresis onto nitrocellulose membrane (Millipore). After electrophoresis, the membrane was incubated with 5% powdered skimmed milk (in tris-buffered saline containing 0.05% Tween 20) overnight. The blocked membrane was incubated sequentially with anti-PBP1 antibody at a dilution of 1:2,000 for 2 h, and then alkaline phosphatase-conjugated goat anti- rabbit IgG-HRP (Sigma-Aldrich, St. Louis, MO, United States) at a dilution of 1:1,000 for 1 h. Immunoreactive bands were detected using 5-bromo-4-chloro-3-indolyl phosphate (BCIP, 0.15 mg/ml) and nitrotetrazolium blue chloride (NBT, 0.3 mg/ml) at a ratio of 1:2.

### Fluorescence Binding Assays

*N*-phenyl-1-naphthylamine (1-NPN) was selected for use as a selectively fluorescent probe to measure the affinity of ligand binding to recombinant EhipPBP1 (Yin et al., 2012; Zhong et al., 2012). Seven pheromone and analog compounds were a gift from Professor Zhang Jintong of Shanxi Agricultural University (**Table 1**) and were >97% pure. A fluorescence binding assay was performed on a multiscan Spectrum Molecular Device SpectraMax i3 (Thermo Scientific, Wilmington, DE, United States) with an excitation wavelength of 337 nm and recording of emission spectra between 380 and 520 nm. Parameter selection was such that the slit widths for both excitation and emission were 10 nm. Spectra were recorded using high-speed scanning. 2 μM solution of EhipPBP1 was prepared in 20 mM Tris-HCl buffer (pH 7.4), and the ligands were dissolved in chromatographically pure methanol as 1 mM stock solutions. The affinity of EhipPBP1 for the labeled probe was determined by adding aliquots of 1-NPN stock solution to give final concentrations of 2–20 μM. The affinity of EhipPBP1 for the different ligands was estimated by competitive binding assays with both 1-NPN and EhipPBP1 at 2 μM, and final concentrations of seven competitive pheromone and analog compounds in the range of 2–20 μM. To determine dissociation constants, intensity values corresponding to maximum fluorescence emission were plotted against free ligand concentrations. Assuming that the protein was 100% active and that the stoichiometric ratio between protein and ligand was 1:1 at saturation, the bound ligand was determined from the fluorescence intensity values. EhipPBP1 binding with every component was replicated six times. The curves were then linearized using Scatchard plots to calculate *K_1-NPN_* values. Dissociation constants of the competitors (*K*_i_) were calculated from the corresponding IC_50_ values by the following equation: *K*_i_ = [IC_50_]/(1 + [1–NPN]/*K_1-NPN_*), where [IC_50_] was the concentration of a competitor that caused a 50% reduction in the fluorescence intensity, [1–NPN] represented the free concentration of 1–NPN, and *K_1-NPN_* represented the dissociation constant of the complex of protein with 1–NPN (Campanacci et al., 2001).

**Table 1:** Binding ability of recombinant EhipPBP1 to seven compounds.

Ligand	Structural formula	Highest combined rate (%)	IC_50_	*K*_d_
E3-14:Ac	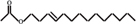	89.404	2.000	1.575 ± 0.210
Z3-14:Ac	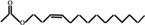	–	–	–
E9-14:Ac	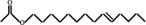	53.100	8.000	6.299 ± 0.521
Z7-14:Ac	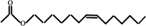	86.403	1.500	1.181 ± 0.012
Z9-14:Ac	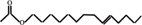	37.932	–	–
Z3-14:OH	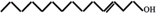	56.492	3.000	2.362 ± 0.201
Z7-14:OH	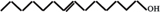	62.272	4.000	3.150 ± 0.321

*Best combined rate refer to EhipPBP1 binding ligand in maximum divide total content of ligand.*

## Results

### Coding and Amino Acid Sequences

**FIGURE 1:**
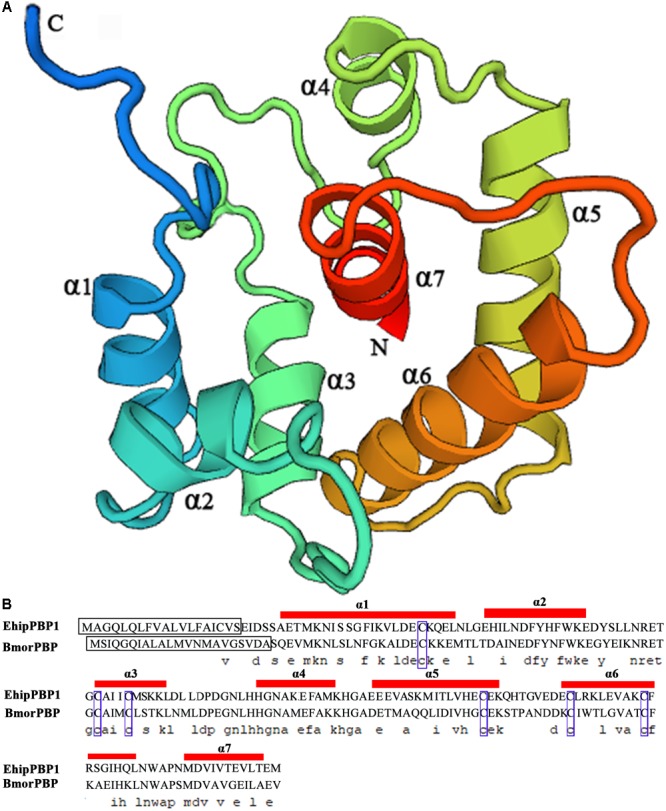
Three-dimensional structure of EhipPBP1. **(A)** 3D structure of EhipPBP1 was used SWISS MODEL online tools (http://swissmodel.expasy.org/) and based on NMR Structure of Pheromone binding protein from *B. mori* (accession number: NP_001037494.1) as model (Horst et al., 2001a). **(B)** Alignment of EhipPBP1 with BmorPBP (accession number: NP_001037494.1). The seven α-helices were as follow, Ala25–Leu46 (α1), Glu50–Lys61 (α2), Cys73–Lys81 (α3), His93–Lys102 (α4), Glu107–Lys122 (α5), Glu130–Gln147 (α6), and Met154–Thr163 (α7); the black boxes refer to signal peptide; the six blue boxes refer to six conserved cysteines.

The coding sequence was identical to the previously identified PBP1 sequence in the *E. hippophaecolus* transcriptome. The EhipPBP1 ORF was 498 bp, and it encoded 166 amino acids, with a predicted size of 18.65 kDa and an isoelectric point of 4.01, and a 19 amino acid N-terminal signal peptide. The full-length ORF sequence was submitted to GenBank, with the accession number KX655931. A prediction of the three-dimensional structure of EhipPBP1, made with SWISS MODEL online tools, is shown in **Figure 1**. The QMEAN total score was 2.49. The structure contained seven α-helices: Ala25–Leu46 (α1), Glu50–Lys61 (α2), Cys73–Lys81 (α3), His93–Lys102 (α4), Glu107–Lys122 (α5), Glu130–Gln147 (α6), and Met154–Thr163 (α7). Six cysteine residues were predicted to form three pairs of disulfide bonds, Cys42–Cys77, Cys73–Cys131, and Cys120–Cys140, connecting α1–α3, α3–α6, and α5–α6, respectively, which corresponded to the known structure of PBPs.

### Expression and Purification of Recombinant EhipPBP1

Induction with IPTG resulted in a protein band on SDS-PAGE at about 16 kDa, consistent with the expected size (Supplementary Figure S1). After lysis and centrifugation of the cells, the recombinant protein was mainly located in the sediment, indicating that EhipPBP1 was expressed as an inclusion body. The recombinant protein was purified by affinity chromatography, and then denatured and renatured to obtain soluble purified protein.

### Gene and Protein Expression Pattern Analysis of EhipPBP1

**FIGURE 2:**
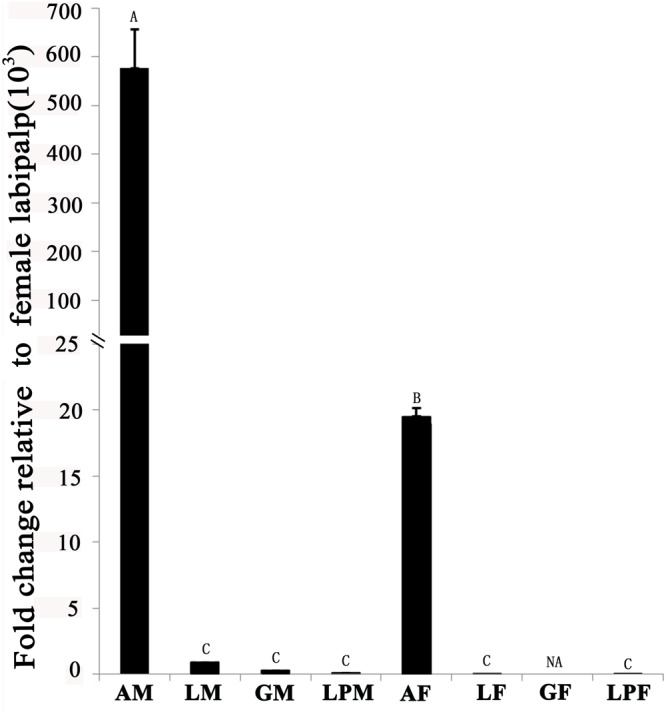
Pheromone-binding protein EhipPBP1 transcript levels in four tissues of both sexes of *E. hippophaecolus*. AM, male antennae; LM, male legs; GM, male external genitals; LPM, male labipalps; AF, female antennae; LF, female legs; GF, female external genitals; LPF, female labipalps. Actin was used as the reference gene to normalize target-gene expression. Error bars show standard errors, and columns with different capital letters (A, B, C) are significantly different from each other, at *p* < 0.01. NA refer to no expression.

Tissue-expression profile of the EhipPBP1 gene (**Figure 2**) indicated that it was most highly expressed in the antennae compared with other tissues, and that expression in male antennae was significantly higher than in female antennae. No difference in expression was observed between the different non-antennal tissues, with the exception of the female external genitals, in which there was no detectable expression.

**FIGURE 3:**
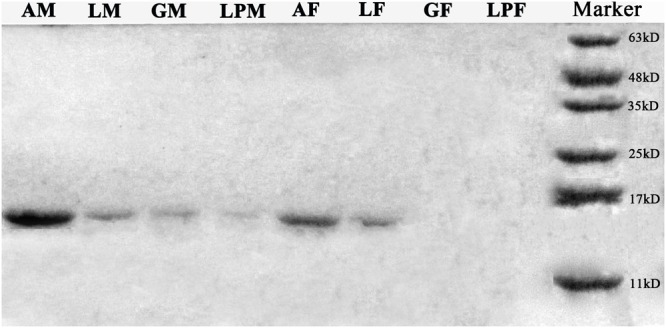
EhipPBP1 protein expression in insect tissues in both sexes of *E. hippophaecolus*. AM, male antennae; LM, male legs;; GM, male external genitals; LPM, male labipalps; AF, female antennae; LF, female legs; GF, female external genitals; LPF, female labipalps. Marker, protein molecular-weight markers (from bottom) 11, 17, 25, 35, 48, and 63 kD.

Western blots of protein extracts from four tissues in male and female *E. hippophaecolus* showed that EhipPBP1 was expressed in male antennae, labipalps, legs, and external genitals, as well as female antennae and legs. According to the gradation of stripe color, PBP1 expression was highest in the antennae, particularly in males; apart from the antennae, expression was highest in the legs (**Figure 3**).

### Fluorescence Binding Assays

**FIGURE 4:**
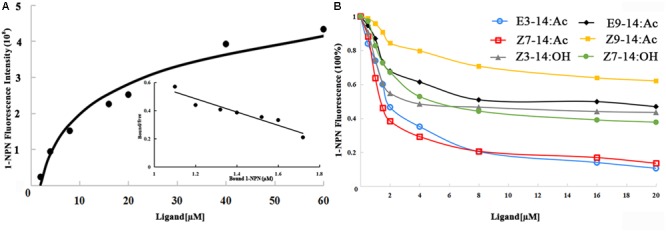
Fluorescence competitive binding assay of EhipPBP1. **(A)** The binding curve and Scatchard analysis of EhipPBP1 and 1-NPN. **(B)** The binding curve of EhipPBP1 and 1-NPN in competition with pheromone components and analogs. Pheromone components: E3-14: Ac and Z7-14: Ac, Pheromone analogs: Z3-14:Ac, E9-14:Ac, Z9-14:Ac, Z3-14:OH, and Z7-14:OH.

The 1-NPN probe, in isolation, produced weak fluorescence on excitation at 337 nm. With the addition of EhipPBP1 protein, the emission spectrum of 1-NPN shifted from 480 to 400 nm, with a considerable, 1-NPN-dose-dependent increase in fluorescence intensity (**Figure 4**). Use of the Scatchard method to linearize the curve resulted in a dissociation constant (*K_1-NPN_*) of 3.7 ± 0.06 μM. In competitive binding assays with 1-NPN and elements of the female pheromone of *E. hippophaecolus*, the calculated *K*_d_ values were 1.58 ± 0.21 μM for E3-14:Ac and 1.18 ± 0.01 μM for Z7-14:Ac, indicating a greater affinity of EhipPBP1 for Z7-14:Ac (**Figure 4** and **Table 1**). EhipPBP1 also demonstrated binding to structural analogs of the female pheromone. For E9-14: Ac, the *K*_d_ was 6.30 ± 0.52 μM, but binding to Z9-14:Ac do not reach 50% in the range of test concentrations, and so we could not calculate the *K*_d_. No binding was detected with the structural analog Z3-14:Ac. Binding to 14 carbon-residue alcohols gave *K*_d_ values of 2.36 ± 0.20 μM for Z3-14: OH and 3.15 ± 0.32 μM for Z7-14:OH (**Figure 4** and **Table 1**).

## Discussion

Our results demonstrated that EhipPBP1 was highly expressed in the antennae of the seabuckthorn carpenterworm moth *E. hippophaecolus* relative to other tissues. Expression was significantly higher in the male antennae than the female antennae, and was significantly lower in all other tissues examined. This result was consistent with the antennal bias of PBP expression that was previously observed in *P. xylostella*, in which the expression of the PBP1 and PBP3 genes is also significantly higher in males than in females (Sun et al., 2013). Similarly, in *A. ipsilon*, expression of three PBP genes is significantly higher in the antennae than in other tissues, and expression in male antennae is significantly higher than in female antennae (Gu et al., 2013). However, this gender bias is not universally observed. For example, in *Cydia pomonella*, PBP1 is specifically expressed in the antennae, but without significant differences between males and females (Tian and Zhang, 2016).

Our western blotting results for EhipPBP1 protein were consistent with the quantitative PCR results. Similar consistency has previously been shown in *C. pomonella* (Tian and Zhang, 2016), and also in *Cnaphalocrocis medinalis*, in which CmedPBP4 protein and mRNA were shown to be specifically expressed in antennae, with a significant male bias (Sun et al., 2016). In the present study, both PCR and western blotting results indicated that the highest non-antennal level of expression of EhipPBP1 was in the legs. In the seabuckthorn carpenterworm, searching and mating involve a “female down, male up” posture, and the trichoid sensillum also located in legs (Hu et al., unpublished), all suggest that the legs might participate in pheromone identification, and thereby facilitate mating.

EhipPBP1 expression in female antennae was higher than in non-antennal tissues. Electroantennogram (EAG) experiments have shown that female moths can detect their own sex-pheromone signals and respond to them (Schneider et al., 1998; Fan et al., 2003). It has been suggested that expression of PBPs in the antennae of female insects may be associated with the feedback regulation of the release of female pheromones (Vogt, 2002). According to this mechanism, female insects in the field need to be able to detect sex pheromones released by females of the same species, to determine whether to release pheromones to attract males. However, EhipPBP1 expression was higher in male legs and labipalps than in females, and notably EhipPBP1 was expressed in male external genitals, but not in females, suggesting that, when males and females mate, the male external genitals can function in the identification of pheromones, but the female external genitals cannot.

We measured the affinity of EhipPBP1 for two components of *E. hippophaecolus* pheromone, and found it was higher for Z7-14:Ac than for E3-14:Ac, but without significant (Supplementary Table S1). This result is consistent with that of a previous report in which three PBPs of *P. xylostella* were found to have different affinities for pheromone components (Sun et al., 2013). Similarly, with two analogs of sex-pheromone components, the affinity of EhipPBP1 binding to E9-14: Ac was much higher than to Z9-14:Ac, Z7-14:Ac and E3-14:Ac, suggesting that EhipPBP1 cannot distinguish between molecules that differ only in carbon double-bond position and *cis*/*trans* structures. By contrast, EhipPBP1 binding was similar with both of the 14-carbon alcohols that were tested. Because ORs are directly responsible for activation of olfactory neurons and conveyance of olfactory information to the brain, PBPs have limited ability in odor discrimination and some downstream components, such as ORs and olfactory receptor neurons (ORNs) are involved in the specificity of pheromone reception (Sun et al., 2013; Rahman and Luetje, 2017; Zhang et al., 2017).

We demonstrated, by measurement of mRNA and protein levels in four tissues in the seabuckthorn carpenterworm, which the highest expression of EhipPBP1 occurred in the antennae, with expression in males higher than in females. Recombinant EhipPBP1 bound to pheromone components and their analogs, and to seabuckthorn volatiles. Our results suggested that EhipPBP1 functions in pheromone recognition in the antennae, especially in male. Therefore, EhipPBP1 could influence mating. This research supports EhipPBP1 can serve as a potential molecular target for the development of eco-friendly pest management strategies against outbreaks of the seabuckthorn carpenterworm. Because of the complexity to feed *E. hippophaecolus* during the 4 years of its life cycle, we are exploring the way to rear the insect on an artificial diet, then we are planning to use the RNAi technique to demonstrate the functions of EhipPBP1 in this organism.

## Author Contributions

PH carried out the molecular genetic studies, performed all the experiments, and drafted the manuscript. CG collected almost all samples and participated in expression analysis by fluorescence quantitative real-time PCR, western blotting, and fluorescence binding assays experiments. JT, YL, and SZ designed and conceived of the study. JT and YL also helped to draft the manuscript. All authors read and approved the final manuscript.

## Conflict of Interest Statement

The authors declare that the research was conducted in the absence of any commercial or financial relationships that could be construed as a potential conflict of interest.

## Footnotes

1 http://bioinfo.ut.ee/primer3-0.4.0/

2 http://www.ncbi.nlm.nih.gov/gorf/gorf.html

3 http://www.cbs.dtu.dk/services/SignalP/

4 http://www.expasy.org/

5 http://swissmodel.expasy.org/
