# Supplementary material for: Pheromone Binding Protein EhipPBP1 Is Highly Enriched in the Male Antennae of the Seabuckthorn Carpenterworm and Is Binding to Sex Pheromone Components
Source: Front Physiol. 2018 Apr 27;9:447. doi: 10.3389/fphys.2018.00447 (PMC5934486; doi:10.3389/fphys.2018.00447)
Supplement: Supplementary file 1 [file Table_1.DOCX]

**Pheromone Binding Protein EhipPBP1 Is Highly Enriched in the Male Antennae of the Seabuckthorn Carpenterworm and Binding to Sex Pheromone Components**

**Ping Hu, Chenglong Gao, Shixiang Zong, Youqing Luo* , Jing Tao***

*** Correspondence:**  You-Qing Luo and Jing Tao

E-mail: [youqingluo@126.com](mailto:youqingluo@126.com), [taojing1029@hotmail.com](mailto:taojing1029@hotmail.com)

**Supplementary Table 1 The multiple comparisons of K*_d_* value of fluorescence competitive binding assay of EhipPBP1**

| (I) CHEM | | (J) CHEM | | Mean Difference (I-J) | significance | 95% confidence interval | |
| --- | --- | --- | --- | --- | --- | --- | --- |
|  |  |  |  |  |  | lower limit | upper limit |
| dimension2 | E3-14:Ac | dimension3 | E9-14:Ac | -4.723333^*^ | .000 | -5.27362 | -4.17305 |
|  |  |  | Z7-14:Ac | .394667 | .141 | -.15562 | .94495 |
|  |  |  | Z3-14:OH | -.787000^*^ | .010 | -1.33729 | -.23671 |
|  |  |  | Z7-14:OH | -1.560000^*^ | .000 | -2.11029 | -1.00971 |
|  | E9-14:Ac | dimension3 | E3-14:Ac | 4.723333^*^ | .000 | 4.17305 | 5.27362 |
|  |  |  | Z7-14:Ac | 5.118000^*^ | .000 | 4.56771 | 5.66829 |
|  |  |  | Z3-14:OH | 3.936333^*^ | .000 | 3.38605 | 4.48662 |
|  |  |  | Z7-14:OH | 3.163333^*^ | .000 | 2.61305 | 3.71362 |
|  | Z7-14:Ac | dimension3 | E3-14:Ac | -.394667 | .141 | -.94495 | .15562 |
|  |  |  | E9-14:Ac | -5.118000^*^ | .000 | -5.66829 | -4.56771 |
|  |  |  | Z3-14:OH | -1.181667^*^ | .001 | -1.73195 | -.63138 |
|  |  |  | Z7-14:OH | -1.954667^*^ | .000 | -2.50495 | -1.40438 |
|  | Z3-14:OH | dimension3 | E3-14:Ac | .787000^*^ | .010 | .23671 | 1.33729 |
|  |  |  | E9-14:Ac | -3.936333^*^ | .000 | -4.48662 | -3.38605 |
|  |  |  | Z7-14:Ac | 1.181667^*^ | .001 | .63138 | 1.73195 |
|  |  |  | Z7-14:OH | -.773000^*^ | .011 | -1.32329 | -.22271 |
|  | Z7-14:OH | dimension3 | E3-14:Ac | 1.560000^*^ | .000 | 1.00971 | 2.11029 |
|  |  |  | E9-14:Ac | -3.163333^*^ | .000 | -3.71362 | -2.61305 |
|  |  |  | Z7-14:Ac | 1.954667^*^ | .000 | 1.40438 | 2.50495 |
|  |  |  | Z3-14:OH | .773000^*^ | .011 | .22271 | 1.32329 |

Note：* The significance level of the mean difference was 0.05. The results of comparative analysis of K*d* value were assessed by a one-way nested analysis of variance (ANOVA), followed by Tukey’s honestly significance difference (HSD) tests implemented in SPSS Statistics 18.0.
