# Supplementary material for: Pheromone Binding Protein EhipPBP1 Is Highly Enriched in the Male Antennae of the Seabuckthorn Carpenterworm and Is Binding to Sex Pheromone Components
Source: Front Physiol. 2018 Apr 27;9:447. doi: 10.3389/fphys.2018.00447 (PMC5934486; doi:10.3389/fphys.2018.00447)
Supplement: Supplementary file 2 [file Image_1.PDF]

Supplementary Material

Pheromone Binding Protein EhipPBP1 Is Highly Enriched in the Male Antennae of the Seabuckthorn Carpenterworm and Binding to Sex Pheromone Components

Ping Hu, Chenglong Gao, Shixiang Zong, Youqing Luo* , Jing Tao*

*** Correspondence:** You-Qing Luo and Jing Tao

E-mail: youqingluo@126.com, taojing1029@hotmail.com

Supplementary Figure 1

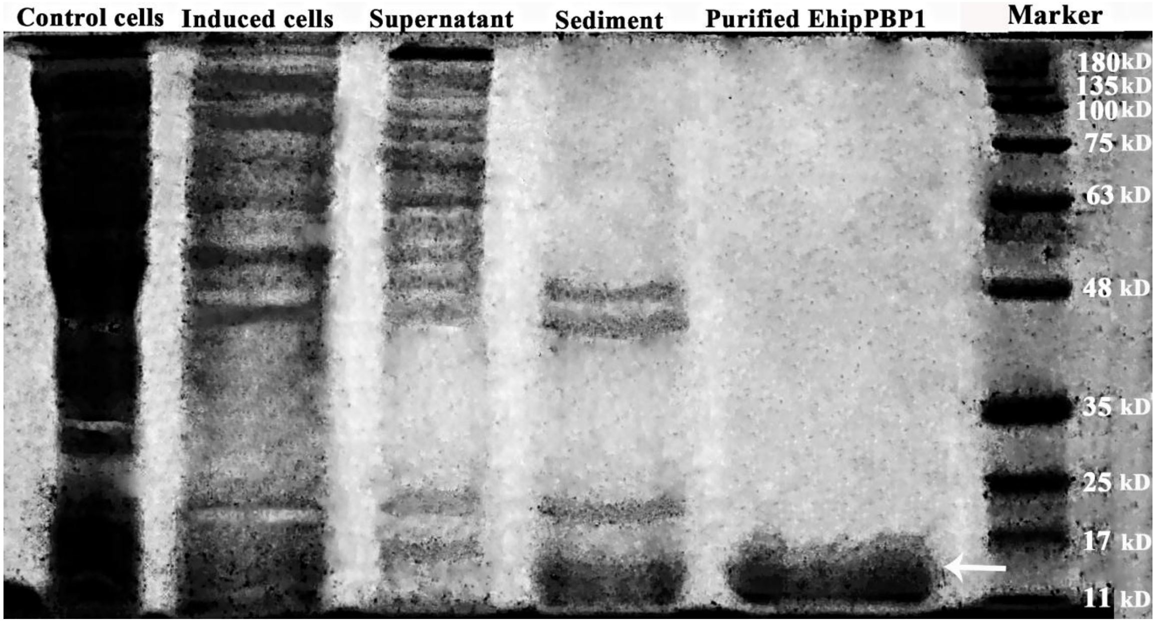

Supplementary Figure 1 Expression of recombinant EhipPBP1 and analysis by SDS-PAGE.

Control cells containing pET30a-EhipPBP1 recombinant expression vector, not induced with IPTG; Induced cells containing pET30a-EhipPBP1 induced with IPTG; Supernatant and Sediment pellet were obtained by centrifugation lysate of pET30a-EhipPBP1which contained cells inducted with IPTG. Purified EhipPBP1, EhipPBP1 purified from inclusion bodies, denatured, and renatured. Marker, protein molecular weight standards. Arrow indicates target protein.
